# Consumer‐Led Codesign of an Effective Online Consumer and Community Involvement Audit Tool

**DOI:** 10.1111/hex.70249

**Published:** 2025-03-31

**Authors:** Asvini K. Subasinghe, Jo Wilkie, Rebekah Puls, Tanya Tuffrey, Jacqueline A. Boyle

**Affiliations:** ^1^ Eastern Health Clinical School Monash University Box Hill Victoria Australia; ^2^ Western Australian Health Translation Network Nedlands Western Australia Australia; ^3^ Perron Institute for Neurological and Translational Science Nedlands Western Australia Australia

**Keywords:** community involvement, consumer participation, equity, evaluation audit, health, literacy

## Abstract

**Introduction:**

There is evidence that consumer and community involvement (CCI) improves the quality and outcomes of health research. However, there is currently limited support for organisations to plan, implement and evaluate CCI activity. We aimed to codesign an audit tool that will enable organisations, researchers, community members and funders to measure the extent and nature of CCI in their respective settings. The tool was based on the Western Australian Health Translation Network's CCI Handbook.

**Methods:**

We offered optional online workshops to inform participants about the study (total *n* = 9). Following this, we obtained feedback on the appropriateness of the Handbook and audit tool (*n *= 11). Finally, using Qualtrics surveys, we assessed the quality and effectiveness of the tool in evaluating CCI with 10 researchers, 6 funders, and 1 community member (total *n* = 17) sourced from networks within Monash University and the Perron Institute for Neurological and Translational Science.

**Results:**

Overall participants had a positive response to the audit tool and believed it had value in quantifying direct implementation of CCI in their workplaces. Gaps identified included the need to address health and research literacy of community members, culturally responsive approaches when working with community members from migrant backgrounds and an identified need for visual and digital resources.

**Conclusions:**

Application of the tool enabled participants to identify their respective CCI strengths, weakness and opportunities for improving the meaningful involvement of community members and community in their research activities and to support equity in these processes.

**Patient or Public Contribution:**

The public participated in workshops providing feedback on the general structure of the audit tool as well as testing it within their research projects.

## 1 Introduction

The benefits of Consumer and Community Involvement (CCI) are increasingly recognised globally by governments, research bodies and health organisations that are fostering the involvement of community members through legislation, policies and financial investment [1]. Other terminology used interchangeably with CCI include consumer consultation, participation, public engagement, codesign, patient‐centred/patient‐oriented research, collaboration, patient and public involvement, and partnership [2]. There is evidence that CCI improves the calibre and quality of the research and, by extension, improves health outcomes [1]. As primary stakeholders (funders, users and beneficiaries), community members have an inherent right to be involved in health and medical research and should be encouraged, supported and given opportunities to do so – at every level and across all aspects of research.

Globally, there are a number of organisations dedicated to improving CCI in translational health research. In Canada, the Ontario SPOR SUPPORT Unit comprises 14 health research centres and 8 research initiatives that connect researchers, patients and other collaborators in patient‐oriented research [3]. In the UK, the National Institute for Health and Care Research has established initiatives such as a Research Champions programme which involves an interactive training programme and resources for patients and researchers [4]. In the US, the Patient‐Centred Outcomes Research Institute (PCORI) funds patient‐centred research to help them understand which medical services and treatments are the most effective to improve their quality of life [5].

The Australian Health Research Alliance (AHRA) is committed to improving CCI and, in 2020, together with the Consumer Health Forum, released a Position Statement on CCI in health research describing the guiding principles for CCI [6]. In 2018, AHRA coordinated a nation‐wide CCI audit to assess the extent and nature of CCI in Australia [7]. The Audit involved an online survey of 868 people across Australia; a review of over 200 research papers; and a comparison of CCI in Australia with leading CCI initiatives overseas [7]. The Audit resulted in four priority recommendations: Developing minimum standards for good practice in CCI which should be a practical and complementary resource to the NHMRC Statement on CCI in Health and Medical Research; facilitating sharing of existing resources and expertise to support CCI in translational research; increased funding for research projects to identify how to increase CCI in health and medical research and measure its impact as well as assessing the efficacy of any existing CCI tools and resources; and initiating formal alliances between AHRA and leading organisations promoting CCI globally [6].

The Western Australian Health Translation Network has developed a CCI Handbook [8] as part of an AHRA [9] National CCI Initiative funded by the Medical Research Future Fund, with additional support from the Western Australian Government. The Handbook was co‐designed by community members, researchers, and research organisations with input from funders and policy‐makers. It complements the rich suite of consumer involvement research, frameworks, policies and toolkits that have been developed by governments and organisations Australia‐wide and internationally [10] by addressing a widely acknowledged barrier to embedding CCI: where to start and how to measure how well CCI is embedded in your research [11]. There are audit tools that assess CCI readiness, developed by Monash Partners [12] and a consumer participation audit tool developed by the Network of Alcohol and Other Drug Agencies [13] for those engaging in research related to alcohol and drugs. However, these tools are not generalisable to all health research activities, are not applicable to researchers, funders, organisations and consumers themselves. The aim of this study was to codesign and codevelop an audit tool that enables organisations, researchers, community members and funders to measure the extent and nature of CCI in their respective settings. Two sites involved in health and medical research participated in this study: The Perron Institute for Neurological and Translation Research in Perth and the Eastern Health Clinical School at Monash University Melbourne. At each site, organisations, researchers, community members and funders were involved.

## 2 Materials and Methods

Ethics: Ethics approval was sought from the Monash University Human and Research Ethics Committee (Project ID: 38473) to conduct the study across both sites (WA and Vic).

### 2.1 Recruitment and Remuneration

Organisations, researchers and community members were recruited through purposive sampling [14] by emailing contacts within internal networks, while organisations who have funded research at Eastern Health/Eastern Health Clinical School and the Perron Institute were approached directly. All participants were provided with an explanatory statement and an email invite ‐ consent was implicit upon acceptance of invitations to join online workshops. Community members received $40 per hour for time committed to the project based on the Safer Care Victoria guidelines [15].

### 2.2 Phase 1: Development of Audit Tool

The audit tool was developed by two co‐authors based on the current CCI Handbook to assess the following key areas:

1. Organisational culture with respect to CCI.
2. Current consumer involvement as defined in the Handbook.
3. Awareness and understanding of CCI benefits and the many types of involvement.
4. The extent to which CCI commitment is reflected in organisational policies and practices.
5. The level of cash and in‐kind investment in CCI.
6. Clarity about motivations and expectations.
7. Alignment between CCI commitment, aspirations and resources.
8. Real and perceived barriers, enablers, challenges.
9. Potential mechanisms for measuring CCI output, outcomes and impact.

### 2.3 Review of the Audit Tool and Handbook

Across both sites, two optional online information sessions, comprising a total of 9 participants were conducted to inform stakeholders about the purpose of the study and background of the Handbook and working draft audit tool. The appropriateness of the tool was then discussed afterwards in separate online workshops with a total of 11 participants (4 researchers, 2 funders, 2 research managers and 3 community members), the latter of whom belonged to Chinese backgrounds. Participants were provided the option to deliver written feedback via email or during discussions held online. The outcomes from this phase are presented in the Results section.

### 2.4 Phase 2: Applying the Audit Tool to Actual Research Activity

The completed tool was then applied to practice by 17 participants. Feedback was obtained via an online version of the audit tool questions, using the Qualtrics platform with one added question asking: ‘Any other comments about the Steps and/or Resources’ (If you have no other comments please type N/A).

Participants were required to nominate their role as either a Research administrator, Researcher, Consumer or Funder and then were provided a link to the WAHTN Handbook. For each step in the Handbook, relevant to their role, participants were asked to make a personal assessment based on their own experience and what knowledge and information they had at the time of completing the survey about how well they had actioned steps outlined in the Handbook. Participants could respond to each step stating that they had either undertaken this step, am currently undertaking this step, am not undertaking this step and am unsure.

Reflexive thematic analysis [16] was conducted by AKS to extract key descriptive themes from the discussions that arose from the online workshops and was reviewed by J.A.B. The key descriptive themes were then systematised in a matrix, and similarities, differences and contradictions were examined using Microsoft Excel. Analytical themes were then developed to answer our questions around how we can improve the audit tool.

## 3 Results

The audit tool that was co‐developed addressed nine key areas mentioned above and links to relevant areas in the Handbook (Appendix 1).

### 3.1 Phase 1: Review of the Audit Tool and Handbook (*N* = 11)

Overall participants in this phase reported that CCI was an important component of research but that it was difficult to implement at times. The concept of an audit tool was perceived to be useful in supporting CCI, and the way it linked to the relevant sections in the Handbook was beneficial. Whilst the aim of the project was to design and evaluate an audit tool, there was also feedback given on the Handbook and about the implementation of CCI for organisations, researchers and community members. These results are presented together here.

A general theme reported by participants at this phase was that there was a lack of equity and health and research literacy within both the audit tool and the Handbook. For example, describing the way researchers can communicate to their intended audience in simple to understand terms and how to reach community members from migrant backgrounds. This is addressed in further detail below.

#### 3.1.1 General Feedback on the Handbook

Overall, participants found the Handbook informative and thorough but quite dense.

The Handbook was ‘organised well, but a big booklet. Think about ways to break down the information and make it more accessible – broken down into modules will be excellent’ [Researcher].

Case studies on how CCI has been implemented well were seen as beneficial to participants in thinking about how to implement CCI in their own research. Participants also requested more examples of resources/hyperlinks related to Policy as the information provided in the Handbook was not comprehensive. For example, ‘Learning modules with a speaker with it and visuals‘ (would be helpful for everyone using the handbook, including researchers and community members) [Researcher].

#### 3.1.2 Implementing CCI at an Organisational Level

Participants also reported a disconnect between the strategic plan of their organisation and goals for CCI and its implementation. They identified the need for clear communication from their organisation as to whether CCI is a strategic goal, with clear expectations for researchers including an indication of how much time, proportionate to their work schedules, should be spent in implementing CCI in their research.

Implementation of the use of the Handbook and integration of CCI within an organisation would be important to support researchers and managers in their work.

#### 3.1.3 General Feedback on the Audit Tool

In general, participants reported that the audit tool appeared to be useful in directing their efforts in CCI.

Tool is quite clear to evaluate where you are and what you should do to improve CCI in your research.

[Researcher]

#### 3.1.4 Enhancing Reach and Access of Audit Tool

To ensure that community members from migrant backgrounds can actively engage with, and access, this tool, community members suggested sharing using a number of approaches that reach community (e.g., advertising on WeChat for those of Chinese background) and better‐equipping community members from priority groups to engage with research organisations in the first instance:

It was suggested that we ‘Prepare a set of short questions for community members of migrant backgrounds to use when approaching organisations and whether you can just see whether you are qualified… [to work with them]’ [Consumer].

#### 3.1.5 Equitable Messaging Across the Handbook and Audit Tool

To improve engagement between researchers, organisations and community members from various ethnic backgrounds, a tailored culturally responsive approach needs to be undertaken to ensure optimal engagement between each stakeholder. Specifically, ‘Messaging/communicating this information to different groups and different levels within an organisation will have to be coordinated as its own thing’ [Consumer].

This information will help guide

where a consumer can fit in based on these data as this will grab the community members attention. If they need female Chinese who visit hospital then it's a good fit. Researchers should articulate what's the next steps and future plans and impact of research‐ this information would help the consumer know the impact of the study.

[Consumer]

There is a lack of information on how to engage and work with people from migrant backgrounds. Participants suggested to transpose all resources from the Handbook into a video format and translated into various languages including subtitles. This would make the content more accessible and easier to understand for people from non‐English speaking backgrounds.

Participants suggested that *Resource 2: Types of Involvement* should be transposed into a link to a video on background information with real people.

Additionally, in reference to *Resource 3: Organisations leading consumer involvement*, community members suggested that organisations should consider the following to help both researchers and consumer representatives: (i) publicly show information about the demographic breakdown of the catchment area their research is intending to target and who attends their health services; (ii) provide outward facing information and advertisements for available roles for community members which include information on the impact the work expects to have on research/policy outcomes. This will help community members to assess whether they are appropriately eligible to contribute their skills/experience to the organisation.

### 3.2 Phase 2: Applying the Audit Tool to Actual Research Activity (*N* = 17)

A total of 17 participants (10 researchers, 6 research administrators, and 1 community member) applied the audit tool using their own projects and experience.

The stakeholders who applied the audit tool using their own projects generally found the audit tool to be very useful in evaluating the level of CCI implemented in their research:

This is an outstanding resource that worked best for me when complemented with meeting the WAHTN CCI team in person.

However, there was still some uncertainty around how much of the work schedule needed to be dedicated to CCI:

Still need clarity on how to implement this while still having a sense of proportion with other areas of the business.

The time commitment for researchers needs to be acknowledged and approved by the group Head.

#### 3.2.1 Reflections From Funders

Almost all research administrators reported that their organisations were currently undertaking the necessary steps to establish effective working relationships with community members, taking a proactive approach to managing CCI involvement to ensure its success and creating channels for potential future involvement with community members. Conversely, about half of the research administrators reported that their organisations were not currently undertaking steps to evaluate CCI, develop appropriate proforma, communicating their commitment to CCI to internal and external stakeholders and actioning the CCI policy in day‐to‐day operations.

#### 3.2.2 Reflections From Researchers

Overall, commitment to CCI and adopting guiding principles around CCI were largely implemented by researchers. However, about half of the researchers reported that they were not currently undertaking steps to evaluate their CCI, developing appropriate proforma to facilitate CCI work, concluding CCI involvement appropriately to ensure future involvement, and developing contingency plans around unplanned conclusions of CCI.

#### 3.2.3 Consumer Feedback

Responses from one consumer showed that they were currently undertaking all the required steps around understanding CCI practices and ensuring they are taking appropriate measures to engage with organisations that will facilitate their involvement in CCI research.

### 3.3 Future Recommendations

Through this project, we have successfully completed the critical first stage of the development of a new tool for assessing the extent and nature of CCI within an organisation and within specific research projects. Feedback from community members, researchers, research organisations and funders were largely positive and additional recommendations on cultural modifications, inclusion of equity and consumer research literacy were provided. Our approach to addressing these recommendations is outlined in Appendix 2 as amendments/additions to relevant steps in the Handbook.

A summary of some of the key learnings gained from evaluating the usefulness of this CCI audit tool and what we have now already addressed are as follows:

– Balancing the comprehensiveness and navigability of the tool.
– Balancing local content and external content.
– Need for structural support within organisations so that CCI could be included in workloads.
– Need for adapting the tool for use by communities of different ethnicities, including suggesting resources and organisations that can provide specific guidance.
– Need to provide guidance for organisations to be transparent about the communities they serve and opportunities for involvement.

The Audit Tool is now ready for full testing in diverse health and medical research settings. Once fully designed, the CCI Audit Tool will work in tandem with the CCI Handbook, which provides the step‐by‐step blueprint for embedding CCI in organisations and research. As is already the case with the CCI Handbook, the CCI Audit Tool will be freely available to all through an online platform, including the CCI Knowledge Hub currently being developed by the Australian Health Research Alliance, through its member Research Translations Centres, led by Monash Partners.

It is planned to now test the CCI Audit Tool in three to five diverse health and medical research settings as the final step in its codesign. The two settings that participated in this study have already committed to participate in this final phase.

The CCI Handbook and Audit Tool will be enhanced by the following current CCI initiatives [5]:

▪ The CCI Knowledge Hub: a national online hub and one‐stop shop for all things CCI, including research directories/reviews, resources, tools, training courses, discussion forums, presentations and other relevant information.
▪ The CCI Training courses: training modules aimed at research organisations, researchers, community members and funders currently under development are due for completion in June 2024.

This audit tool was developed to assist the global research and consumer community with the arsenal required to begin embedding CCI in their work and then accurately measuring to what extent they have achieved this goal. As mentioned previously, unlike other tools available, this audit tool is generalisable to all health research activities, and is applicable to all researchers, funders, organisations and consumers themselves. This tool will be easily adaptable for use in the research communities across the US, UK and Canada where it will complement the patient‐centred frameworks and work already being undertaken in these countries [2, 3, 4, 5].

## 4 Conclusions

We show that overall researchers, administrators, community members and funders had a positive response to a CCI audit tool and believe it has a great value in quantifying direct implementation of CCI in their workplaces. Gaps identified included the need to address health and research literacy, culturally responsive approaches when working with community members from migrant backgrounds and an identified need for visual and digital resources. Testing of the tool identified opportunities for more work to be undertaken by stakeholders around evaluating their CCI work and ensuring appropriate proforma are in place to ensure appropriate terms of reference.

## Author Contributions

J.A.B. and J.W. designed the study. A.K.S. wrote the manuscript and analysed data. R.B. and T.T. reviewed the manuscript.

## Ethics Statement

Ethics approval was sought from the Monash University Human and Research Ethics Committee (Project ID: 38473) to conduct the study.

## Consent

All participants provided informed consent to participate in the study.

## Conflicts of Interest

The authors declare no conflicts of interest.

## Supporting information

Appendix 1.

Appendix 2.

## Data Availability

The data that support the findings of this study are available from the corresponding author upon reasonable request.
