# Supplementary material for: Consumer‐Led Codesign of an Effective Online Consumer and Community Involvement Audit Tool
Source: Health Expect. 2025 Mar 31;28(2):e70249. doi: 10.1111/hex.70249 (PMC11958596; doi:10.1111/hex.70249)
Supplement: Supplementary file 1 — Appendix 1. [file HEX-28-e70249-s001.docx]

**Appendix 1: The Consumer and Community Involvement Audit Tool**

1. **The Research Organisation**

Moving from good intentions to good practice


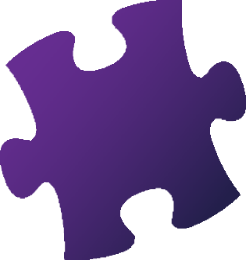

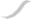

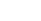

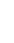


Leadership, a supportive culture and a financial commitment from the organisation are imperative to move CCI from good intentions to good practice. The following steps set up a staged approach staged approach for establishing the leadership, governance and management required for the systemic embedding of Consumer Involvement in organisational policy and practice.

| **Step** | **Description** | **Resources** | *There is documented evidence that the organisation has this in place* | *The organisation is currently working towards putting this in place* | *The organisation does not have this in place* | *To what degree are the resources useful for achieving this step*  *1-5* |
| --- | --- | --- | --- | --- | --- | --- |

**PHASE 1: Commitment**

| **Step 1** | Understand CCI including contemporary thinking, potential benefits and types of involvement through:   - published material - online resources - speaking directly with consumers and/or health consumer organisations | ***Resource 1:*** *Overview of CCI*  ***Resource 2:*** *Types of Involvement*  ***Resource 3:*** *Organisations Leading Consumer Involvement* |  |  |  |  |
| --- | --- | --- | --- | --- | --- | --- |
| **Step 2** | - create and adopt a policy and guiding principles - incorporate CCI into the organisation’s Strategic and Operational Plans - include CCI in all organisational policies | ***Resource 4:*** *CCI Policy for Organisations*  ***Resource 5:*** *Policy Checklist* |  |  |  |  |
| **Step 3** | Communicate your commitment to all key stakeholders: internal and external | ***Resource 6:*** *Communication to Key Stakeholders* |  |  |  |  |

**PHASE 2: Planning and Preparation**

| **Step 4** | | Create the right environment for CCI success where:   - commitment to CCI is understood and shared - everyone is informed, prepared and supported - there is clarity about purpose and process | ***Resource 11:*** *Creating a CCI Culture* |  |  |  |  |  |
| --- | --- | --- | --- | --- | --- | --- | --- | --- |
| **Step 5** | | Action the CCI policy by incorporating it into the day-to-day operations including:   - management - accountability - budget and other resources - types of involvement required/desired | ***Resource 12:*** *Involvement Costs and Considerations* |  |  |  |  |  |
| **Step 6** | | Appoint someone to be responsible for CCI | ***Resource 13:*** *CCI Coordinator Role* |  |  |  |  |  |
|  | |  | | ***Resource 14:*** *Recruiting Consumers* |  |  |  |  |
| **Step 7** | | - recruit consumers - aim to recruit the right person for the right role at the right time - provide a mentor | | ***Resource 15:*** *Interviewing Potential Consumers*  ***Resource 16:*** *Setting up a Consumer Register*  ***Resource 17:*** *Request for Expression of Interest* |  |  |  |  |
|  | |  | | ***Resource 18:*** *The Role of the Mentor* |  |  |  |  |

**PHASE 3: Managing for Success**

| **Step 8** | Clarify the involvement arrangement with a suitable document  e.g. Involvement Agreement, Terms of Reference, Letter of Agreement | ***Resource 21:*** *Committee Terms of Reference*  ***Resource 22:*** *The Involvement Agreement* |  |  |  |  |
| --- | --- | --- | --- | --- | --- | --- |
| **Step 9** | - establish trust and build effective working relationships - aim to ensure that all parties are confident, informed, equipped and empowered to be effective and successful in their roles - provide induction and orientation as early as possible - clarify remuneration process | **Resource 23:** *Induction and Orientation*  **Resource 24:** *Remuneration Claim Form* |  |  |  |  |
| **Step 10** | Take a proactive approach to managing the involvement to ensure its success and minimise problems along the way | ***Resource 25:*** *Managing for Success*  ***Resource 26:*** *Managing for Success* |  |  |  |  |

**PHASE 4: Evaluating the Involvement**

| **Step 11** | - take time to evaluate the involvement to build expertise and provide evidence for continuous improvement - involve all parties in the evaluation – the picture is incomplete without all perspectives | ***Resource 27:*** *Evaluating the Involvement* |  |  |  |  |
| --- | --- | --- | --- | --- | --- | --- |
| **Step 12** | - take time to reflect on the involvement - consider learnings - make changes | ***Resource 28:*** *Reflecting on the Involvement* |  |  |  |  |

**PHASE 5: Concluding the Involvement**

| **Step 13a** | Planned Conclusion:   - acknowledge, respect and value the consumer contribution - celebrate shared success - create channels for potential future involvement - ensure the consumer is appropriately acknowledged on all reports, documents and/or presentations | ***Resource 29:*** *Concluding the Involvement - Planned* |  |  |  |  |
| --- | --- | --- | --- | --- | --- | --- |
| **Step 13b** | Unplanned Conclusion:   - protect any benefits that have been achieved - minimise any adverse impact of an unplanned ending to involvement | ***Resource 30:*** *Concluding the Involvement – Unplanned* |  |  |  |  |

1. **Researchers**

**Getting the most out of the consumer’s involvement**


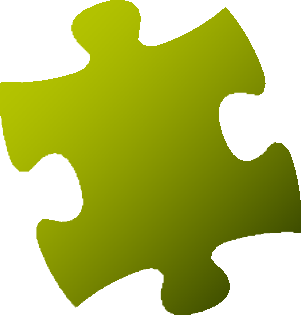

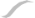

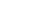


The commitment and buy-in of researchers (including clinicians) is important to the successful involvement of consumers. These steps are designed to support researchers to optimise the involvement of consumers in research projects and programs. Central to the success are:

- the engagement of consumers as early as possible in the development of the research project
- the establishment of a strong positive relationship, and
- a shared understanding of roles and responsibilities amongst all members of the research team

| **Step** | **Description** | **Resource** | There is documented evidence that the researcher team has this in place | The researcher team is working toward putting this in place | The researcher team does not have this in place | To what degree are the resources useful for achieving this step  1-5 |
| --- | --- | --- | --- | --- | --- | --- |

**PHASE 1: Commitment**

| **Step 1** | Understand CCI – latest thinking and evidence, potential benefits and types of involvement | ***Resource 1:*** *Overview of CCI* |  |  |  |  |
| --- | --- | --- | --- | --- | --- | --- |
| **Step 2** | Clarify why consumers are being involved in the research | ***Resource 7:*** *Researchers: Why Involve Consumers in Research* |  |  |  |  |
| **Step 3** | Include Consumer Involvement in all relevant research policies | ***Resource 5:*** *Policy Checklist* |  |  |  |  |
| **Step 4** | Determine how consumers are to be involved in the research, their role and responsibilities | ***Resource 2:*** *Types of Involvement* |  |  |  |  |

**PHASE 2: Planning and Preparation**

| **Step 5** | Create the right environment for CCI success where:   - everyone is informed - there is clarity about the roles and responsibilities of all those involved in the research including the consumer | *Resource 11: Creating a CCI Culture* |  |  |  |  |
| --- | --- | --- | --- | --- | --- | --- |
| **Step 6** | Consider the cash and non-cash resources that will be required | *Resource 12: Involvement Cost and Considerations* |  |  |  |  |
| **Step 7** | Recruit the right consumer for the right task at the right time | ***Resource 14:*** *Recruiting Consumers*  ***Resource 15:*** *Interviewing Potential Consumers*  ***Resource 16:*** *Setting Up a Consumer Register*  ***Resource 17:*** *Request for Expression of Interest* |  |  |  |  |
| **Step 8** | Appoint a research mentor  The consumer is part of the research team. Consider how to include and support them | *Resource 18: The Role of the Mentor* |  |  |  |  |
| **Step 9** | Clarify the involvement arrangement with a suitable document e.g. Involvement Agreement, Terms of Reference, Letter of Agreement | ***Resource 21:*** *Committee Terms of Reference*  ***Resource 22:*** *The Involvement Agreement* |  |  |  |  |
| **Step 10** | Check that all important preparations have been completed to avoid problems during the involvement  Clarify remuneration process | ***Resource 23:*** *Induction and Orientation*  ***Resource 24:*** *Remuneration Claim Form* |  |  |  |  |
| **PHASE 3: Managing for Success** | | | | | | |
| **Step 11** | Use the Involvement Agreement to guide periodic catch-ups and amend the Agreement as necessary | ***Resource 25:*** *Managing for Success* |  |  |  |  |
| **Step 12** | Incorporate the management of the involvement within the day-to-day operations as much as possible so that it does not become an additional impost | ***Resource 26:*** *Managing for Success* |  |  |  |  |

**PHASE 4: Evaluating the Involvement**

| **Step 13** | - consider if/how the involvement has added value to the research - measure experiences, benefits and perceptions - use evaluation results to inform continuous improvement to policy and practice | ***Resource 27:*** *Evaluating the Involvement* |  |  |  |  |
| --- | --- | --- | --- | --- | --- | --- |
| **Step 14** | - take time to reflect on the involvement - consider learnings - make changes | ***Resource 28:*** *Reflecting on the Involvement* |  |  |  |  |

**PHASE 5: Concluding the Involvement**

| **Step 15a** | Planned Conclusion:   - acknowledge, respect and value the consumer contribution - celebrate shared success - create channels for potential future involvement - ensure that the consumer is appropriately acknowledged on all reports, documents and/or presentations | ***Resource 29:*** *Concluding the Involvement – Planned* |  |  |  |  |
| --- | --- | --- | --- | --- | --- | --- |
| **Step 15b** | Unplanned Conclusion:   - protect any benefits that have been achieved - minimise any adverse impact of an unplanned ending to involvement | ***Resource 30:*** *Concluding the Involvement – Unplanned* |  |  |  |  |

1. **Consumers**

**Adding meaningful value to health and medical research**

The voice, experience, knowledge and perspective of the consumer, when harnessed well, can transform the quality and relevance of health and medical research.


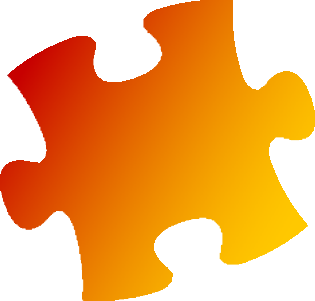

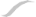

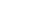

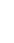


Achieving this requires empowered, informed and well-prepared consumers, able to meet this responsibility and confident that they are the right person in the right role at the right time.

| **Step** | **Description** | **Resource** | **There is documented evidence that the consumer has this in place** | **The consumer is working toward putting this in place** | **The consumer does not have this in place** | **To what degree are the resources useful for achieving this step**  **1-5** |
| --- | --- | --- | --- | --- | --- | --- |

**PHASE 1: Commitment**

| **Step 1** | Understand CCI – latest thinking, potential benefits and types of involvement | ***Resource 1:*** *Overview of CCI* |  |  |  |  |
| --- | --- | --- | --- | --- | --- | --- |
| **Step 2** | Be clear about your motivation for involvement and what you want from it | ***Resource 8:*** *Consumers: Why Get Involved in Research* |  |  |  |  |
| **Step 3** | Consider the variety of ways that you can be involved | ***Resource 2:*** *Types of Involvement* |  |  |  |  |
| **Step 4** | Investigate the health and medical research organisations that involve consumers and which interest you | ***Resource 3:*** *Organisations Leading Consumer Involvement* |  |  |  |  |

**PHASE 2: Planning and Preparation**

| **Step 5a** | | - join consumer health organisations and/or subscribe to their newsletters etc. - register your interest with health research organisations - approach an organisation directly to express your interest | ***Resource 19:*** *Consumers: Approaching an Organisation* |  |  |  |  |
| --- | --- | --- | --- | --- | --- | --- | --- |
| **Step 5b** | | Ensure you make an informed choice about the organisation and research program before you commit | ***Resource 20:*** *Consumers: Assessing the Opportunity* |  |  |  |  |
|  | - ensure that your involvement arrangement is clarified through a suitable document e.g. Involvement Agreement, Terms of Reference, Letter of Agreement - clarify the remuneration process - clarify requirements and expectations, including yours | | ***Resource 21:*** *Committee Terms of Reference* |  |  |  |  |
|  |  |  | ***Resource 22:*** *The Involvement Agreement* |  |  |  |  |
| **Step 6** |  |  | ***Resource 24:*** *Remuneration Claim Form* |  |  |  |  |
|  |  |  |  |  |  |  |  |
|  |  |  |  |  |  |  |  |

**PHASE 3: Managing for Success**

| **Step 7** | - communicate regularly with the researcher or the key organisation contact or mentor - use the Involvement Agreement to guide periodic catch-ups and amend the Agreement as necessary | ***Resource 25:*** *Managing for Success*  ***Resource 26:*** *Managing for Success* |  |  |  |  |
| --- | --- | --- | --- | --- | --- | --- |
|  |  |  |  |  |  |  |

**PHASE 4: Evaluating the Involvement**

| **Step 8** | - participate in the evaluation of the involvement - your contribution is important and will inform how you approach future involvement arrangements | ***Resource 27:*** *Evaluating the Involvement* |  |  |  |  |
| --- | --- | --- | --- | --- | --- | --- |
| **Step 9** | - take time to reflect on the involvement - consider learnings - make changes | ***Resource 28:*** *Reflecting on the Involvement* |  |  |  |  |

**PHASE 5: Concluding the Involvement**

| **Step 10a** | Planned Conclusion:   - acknowledge the opportunity for the involvement - celebrate shared success - create channels for potential future involvement - ensure that your contribution has been appropriately acknowledged on all reports, documents and/or presentations | ***Resource 29:*** *Concluding the Involvement – Planned* |  |  |  |  |
| --- | --- | --- | --- | --- | --- | --- |
| **Step 10b** | Unplanned Conclusion:   - protect any benefits that have been achieved - minimise any adverse impact of an unplanned ending to involvement | ***Resource 30:*** *Concluding the Involvement – Unplanned* |  |  |  |  |

1. **Funders**

**Leading by example**


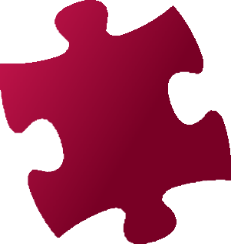

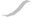

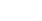


The unique experiences of consumers add immeasurable benefit to the funders’ objectives of improving community health through health and medical research funding. Their involvement ensures the relevance of funding policy, process and criteria. Most importantly, it provides a more holistic context for the responsible and effective allocation of funds.

Funders are powerfully placed to drive Consumer Involvement by mandating it in essential funding criteria. Funders can set the criteria in the confidence that there is readily available, good quality information, resources and tools, including this Handbook, to provide applicants with the necessary support to meet such criteria.

| **Step** | **Description** | **Resource** | *There is documented evidence that the organisation has this in place* | *The organisation is working towards putting this in place* | *The organisation does not have this in place* | *To what degree are the resources useful for achieving this step*  *1-5* |
| --- | --- | --- | --- | --- | --- | --- |

**PHASE 1: Commitment**

| **Step 1** | Understand CCI contemporary thinking, potential benefits and types of Involvement | ***Resource 1:*** *Overview of CCI*  ***Resource 2:*** *Types of Involvement* |  |  |  |  |
| --- | --- | --- | --- | --- | --- | --- |
| **Step 2** | - create and adopt a policy and guiding principles - incorporate CCI into the Strategic Plan - incorporate CCI into the Funding Policy - require applicants to demonstrate CCI as a condition of funding   Involving consumers in this process will ensure effective incorporation of CCI and lead to improved funding outcomes | ***Resource 9:*** *Involving Consumers in Funder Activities*  ***Resource 10:*** *CCI Policy for Funders* |  |  |  |  |
| **Step 3** | Communicate your commitment to all key stakeholders | ***Resource 6:*** *Communication to Stakeholders* |  |  |  |  |

**PHASE 2: Planning and Preparation**

| **Step 4** | | Create the right environment for CCI success where:   - commitment to CCI is understood and shared - everyone is informed, prepared and supported - there is clarity about purpose and process | ***Resource 11:*** *Creating a CCI Culture* |  |  |  |  |  |
| --- | --- | --- | --- | --- | --- | --- | --- | --- |
| **Step 5** | | Action the CCI policy by incorporating it into the day-to-day operations including:   - management - accountability - budget and other resources - types of involvement required/desired | ***Resource 12:*** *Involvement Costs and Considerations* |  |  |  |  |  |
| **Step 6** | | Appoint someone to be responsible for CCI | ***Resource 13:*** *CCI Coordinator Role* |  |  |  |  |  |
| **Step 7** | | Recruit consumers  Aim to recruit the right person for the right role at the right time | | ***Resource 14:*** *Recruiting Consumers*  ***Resource 15:*** *Interviewing Potential Consumers*  ***Resource 16:*** *Setting up a Consumer Register*  ***Resource 17:*** *Request for Expression of Interest* |  |  |  |  |

**PHASE 3: Managing for Success**

| **Step 8** | Clarify the involvement arrangement with a suitable document e.g. Involvement Agreement, Terms of Reference, Letter of Agreement | ***Resource 21:*** *Committee Terms of Reference*  ***Resource 22:*** *The Involvement Agreement* |  |  |  |  |
| --- | --- | --- | --- | --- | --- | --- |
| **Step 9** | - establish trust and build effective working relationships - aim to ensure that all parties are confident, informed, equipped and empowered to be effective and successful in their roles - provide induction and orientation as early as possible - clarify remuneration and payment process | ***Resource 23:*** *Induction and Orientation*  ***Resource 24:*** *Remuneration Claim Form* |  |  |  |  |
| **Step 10** | Take a proactive approach to managing the Involvement.  This will ensure its success and minimise problems along the way. | ***Resource 25:*** *Managing for Success*  ***Resource 26:*** *Managing for Success* |  |  |  |  |

**PHASE 4: Evaluating the Involvement**

| **Step 11** | - take time to evaluate the involvement to build expertise and provide evidence for continuous improvement - involve the consumer - involve the grant recipient as much as you can - this will provide diverse perspectives and build a complete picture | ***Resource 27:*** *Evaluating the Involvement* |  |  |  |  |
| --- | --- | --- | --- | --- | --- | --- |
| **Step 12** | - take time to reflect on the involvement - consider learnings - make changes - communicate the changes | ***Resource 28:*** *Reflecting on the Involvement* |  |  |  |  |

**PHASE 5: Concluding the Involvement**

| **Step 13a** | Planned Conclusion:   - acknowledge, respect and value the consumer contribution - celebrate shared success - create channels and opportunities for potential future involvement | ***Resource 29:*** *Concluding the Involvement – Planned* |  |  |  |  |
| --- | --- | --- | --- | --- | --- | --- |
| **Step 13b** | Unplanned Conclusion:   - protect any benefits that have been achieved - minimise any adverse impact of an unplanned conclusion to the involvement | ***Resource 30:*** *Concluding the Involvement – Unplanned* |  |  |  |  |
