# Supplementary material for: Consumer‐Led Codesign of an Effective Online Consumer and Community Involvement Audit Tool
Source: Health Expect. 2025 Mar 31;28(2):e70249. doi: 10.1111/hex.70249 (PMC11958596; doi:10.1111/hex.70249)
Supplement: Supplementary file 2 — Appendix 2. [file HEX-28-e70249-s002.docx]

Initial Improvements to the CCI Handbook.

Two matters have been initially identified as lacking in the CCI Audit Tool – gender equity and research literacy. Our approach to addressing these is described below as amendments/additions to relevant steps in the Handbook.

Further amendments may be made pending a full review of the survey and preceding workshops.

RESEARCH LITERACY

Organisations – Step 9

| **Step 9** | - establish trust and build effective working relationships - aim to ensure that all parties are confident, informed, equipped and empowered to be effective and successful in their roles - provide training, induction and orientation as early as possible, including training in research literacy - clarify remuneration process | **Resource 23:** *Induction and Orientation*  **Resource 24:** *Remuneration Claim Form*  **Resource:** *Research Literacy for Consumers* |
| --- | --- | --- |

Researcher – Step 8

| **Step 8** | Appoint a research mentor  The consumer is part of the research team. Consider how to include and support them.  Commit to using plain English as much as is possible.  Ensure that the consumer(s) have access to training in research literacy | ***Resource 18:*** *The Role of the Mentor*  *Resources: Research Literacy for Consumers* |
| --- | --- | --- |

Consumer - Step 5a

| **Step 5a** | - join consumer health organisations and/or subscribe to their newsletters etc. - register your interest with health research organisations - approach an organisation directly to express your interest - consider undertaking some learning or training in research literacy to expand your skills | ***Resource 19:*** *Consumers: Approaching an Organisation*  *Resources: Research Literacy for Consumers* |
| --- | --- | --- |

Funder- Step 9

| **Step 9** | - establish trust and build effective working relationships - aim to ensure that all parties are confident, informed, equipped and empowered to be effective and successful in their roles - provide training, induction and orientation as early as possible, including training in research literacy - clarify remuneration and payment process | **Resource 23:** Induction and Orientation  **Resource 24:** Remuneration Claim Form  Resources: Research Literacy for Consumers |
| --- | --- | --- |

EQUITY AND INCLUSION

1. Organisations – Step 7

|  |  | ***Resource 14:*** *Recruiting Consumers* |
| --- | --- | --- |
| **Step 7** | - develop processes around ensuring researchers can - recruit the right person for the right role at the right time - provide a mentor - consider and address equity and inclusion of groups for whom there are social, cultural, physical, geographic and financial barriers to involvement | ***Resource 15:*** *Interviewing Potential Consumers*  ***Resource 16:*** *Setting up a Consumer Register*  ***Resource 17:*** *Request for Expression of Interest* |
|  |  | ***Resource 18:*** *The Role of the Mentor*  ***Resource: Equity and Inclusion*** |

1. Researchers - Step 7

| **Step 7** | Follow appropriate processes around recruit the right consumer for the right task at the right time.  Consider and address equity and inclusion of groups for whom there are social, cultural, physical, geographic and financial barriers to involvement | ***Resource 14:*** *Recruiting Consumers*  ***Resource 15:*** *Interviewing Potential Consumers*  ***Resource 16:*** *Setting Up a Consumer Register*  ***Resource 17:*** *Request for Expression of Interest*  ***Resource: Equity and Inclusion*** |
| --- | --- | --- |

1. Consumer - Step 5b

| **Step 5b** | Ensure you make an informed choice about the organisation and research program before you commit  Consider whether the organisation makes provision for equity and inclusion of groups for whom there are social, cultural, physical, geographic and financial barriers to involvement | ***Resource 20:*** *Consumers: Assessing the Opportunity*  ***Resource: Equity and Inclusion*** |
| --- | --- | --- |

1. Funder – Step 7

| **Step 7** | Recruit consumers  Ensure organisations and researchers have the processes in place to recruit the right person for the right role at the right time  Consider and address equity and inclusion of groups for whom there are social, cultural, physical, geographic and financial barriers to involvement | ***Resource 14:*** *Recruiting Consumers*  ***Resource 15:*** *Interviewing Potential Consumers*  ***Resource 16:*** *Setting up a Consumer Register*  ***Resource 17:*** *Request for Expression of Interest*  ***Resource: Equity and Inclusion*** |
| --- | --- | --- |

Potential Resources to be included in the CCI Audit Tool

The following resources have been identified as a start point for referencing in the CCI Audit Tool. These and other existing resources will be reviewed and considered for inclusion in the revised CCI Audit Tool. Where appropriate a bespoke resource may be created to assist users of the CC Audit Tool to complete the relevant step.

1. **ACCESS AND EQUITY POLICY**

Australian Government (Community Grants Hub Resource)

1. <https://www.communitygrants.gov.au/sites/default/files/documents/2022-06/424-access-equity-download-3.pdf>

Australian Clinical Trials Alliance

1. <https://clinicaltrialsalliance.org.au/resource/recommendations-to-improve-cultural-and-linguistic-diversity-in-clinical-trials/>
2. <https://involvementtoolkit.clinicaltrialsalliance.org.au/toolkit/understanding/diverse-and-inclusive-involvement/>
3. **RESEARCH LITERACY**

University of Sydney

1. Ask Share Know. <http://www.askshareknow.com.au/index.html>.)

Australian Safety and Quality

1. Goals for Health Care Partnering with Consumers: Action Guide. <http://www.safetyandquality.gov.au/wp-content/uploads/2012/08/3-Partnering-withconsumers.pdf>.)

Cancer Australia

1. Consumer Learning – Consumer Involvement in Cancer Cooperative Trial Groups – Information to help consumers understand how they can play a role in research
2. Australia Clinical Trials Alliance

<https://involvementtoolkit.clinicaltrialsalliance.org.au/toolkit/undertaking/training-consumers/>

Other

1. Health literacy universal precautions toolkit45 (Agency for Healthcare Research and Quality): Web site: [www.ahrq.gov/qual/literacy/healthliteracytoolkit.pdf](http://www.ahrq.gov/qual/literacy/healthliteracytoolkit.pdf)
2. Easy does it! Plain language and clear verbal communication training manual89 (Canadian Public Health Association): Web site: [www.cpha.ca/uploads/portals/h-l/easy_does_it_e.pdf](http://www.cpha.ca/uploads/portals/h-l/easy_does_it_e.pdf)
3. Simply put: A guide for creating easy-to-understand materials90 (Centers for Disease Control and Prevention): Web site: [www.cdc.gov/healthcommunication/ToolsTemplates/Simply_Put_082010.pdf](http://www.cdc.gov/healthcommunication/ToolsTemplates/Simply_Put_082010.pdf)
4. Plain language91 (US Government): Web site: [www.plainlanguage.gov/](http://www.plainlanguage.gov/)
